# *Haemophilus influenzae* type b (Hib) seroprevalence in France: impact of vaccination schedules

**DOI:** 10.1186/s12879-021-06440-w

**Published:** 2021-07-30

**Authors:** Eva Hong, Aude Terrade, Mélanie Denizon, Myriam Aouiti-Trabelsi, Michaël Falguières, Muhamed-Kheir Taha, Ala-Eddine Deghmane

**Affiliations:** Invasive bacterial infections unit and National Reference Center for meningococci and Haemophilus influenzae, Institut Pasteur, Paris, France

**Keywords:** *Haemophilus influenzae*, Vaccination, Vaccine failure, Seroprevalence

## Abstract

**Background:**

*Haemophilus influenzae* serotype b (Hib) conjugate vaccine was introduced in France in 1992 as a 3 + 1 scheme at 2, 3, and 4 months (primary vaccination) with a booster at the age of 16–18 months. The vaccination was simplified in 2013 to a 2 + 1 scheme at 2 and 4 months (primary immunization) and a booster at the age of 11 months. The coverage was 95.4% in France at 24 months in 2017. During the period 2017–2019 the number of Hib invasive infections increased with several cases of vaccine failure.

**Methods:**

The numbers and proportions of Hib invasive isolates during the period 2017–2019 were compared and vaccine failure cases were explored. A seroprevalence study was performed by measuring anti-polyribosyl-ribitol phosphate (PRP) IgG concentrations by ELISA among children < 5 years of age at the time of sampling covering the periods of the 3 + 1 or 2 + 1 schemes of Hib vaccination. A collection of residual 232 sera was tested (group 3 + 1 *n* = 130) and (group 2 + 1, *n* = 102) was used.

**Results:**

Anti-PRP IgG concentrations were significantly higher in toddlers of 2 years (median 2.9 μg/ml) in the 3 + 1 group while these concentrations showed a median of 0.58 μg/ml among children in 2 + 1 group. The proportion of children of 2 years of age who achieved 1 μg/ml threshold (56%) was higher in the 3 + 1 group than that observed in the 2 + 1 group (25%). All the detected cases of vaccine failure received the 2 + 1 scheme and anti-PRP IgG levels were less than 1 μg/ml at the admission. However, these levels increased significantly 1 month after the admission suggesting a secondary immune response to the Hib infection.

**Conclusions:**

The simplification of the vaccination to a 2 + 1 scheme seems to reduce the level of anti PRP IgG. Hib antibodies wane rapidly after the 11 months booster and may not be enough to ensure long term protection. Surveillance of cases and monitoring of titres need to be continued to inform future vaccination policy.

## Background

*Haemophilus influenzae* (Hi) is a Gram negative bacterium that can be capsulated or non-capsulated. The structure of the capsule polysaccharides allows dividing the capsulated isolates into six distinct serotypes (Hi a to f) while non-capsulated isolates correspond to non-typeable isolates (NTHi). Hi is a commensal resident of the respiratory and the genital mucosa. Asymptomatic carriage of Hi in healthy children under 5 years is common (27.7%) varying between 73.2% in winter and 26.8% in summer [1]. Hi isolates can be responsible for non-invasive local infections (most frequently respiratory infections) and invasive systemic infections that are defined by the detection of Hi by culture and / or by detection of Hi DNA in a normally sterile site. These invasive infections are mainly septicaemia and meningitis but also arthritis and epiglottitis. The incidence of confirmed cases of invasive infections in Hi in Europe was 0.7 cases per 100,000 in 2016 (0.5 in 2012) with an incidence that varies between 0.0 and 3.6 per 100,000 people in 2016 according to the country (France 0.9 per 100,000 people) [2]. NTHi isolates caused the majority of cases in all age groups (78% of all cases for which typing results were available). Hif caused 11% of all cases with known type. Hib ranked second among these isolates with 6%. However, Hib were the most frequent typeable isolates among children under the age of 5 years [2]. Prior to routine immunization against Hib in the early 1990s, Hib was the most prevalent cause of invasive Hi disease (IHiD) among children. Studies suggested that a serum anti-PRP antibody concentration of at least 0.15 μg/ml and 1.0 μg/ml might correlate with short-and long-term protection respectively from invasive Hib disease [3]. In France, the Hib conjugate vaccine was introduced into the routine childhood immunization program in early 1992 as a 3 + 1 scheme at 2, 3, and 4 months (primary vaccination) and a booster at the age of 16–18 months. The vaccination scheme was simplified in 2013 to a 2 + 1 scheme at 2 and 4 months (primary vaccination) and a booster at the age of 11 months using a hexavalent vaccine (against diphtheria, tetanus, pertussis (acellular vaccine), poliomyelitis, *Haemophilus influenzae* b, and hepatitis B). In 2017, the vaccine coverage was estimated at 95.4% at the age of 24 months (https://www.santepubliquefrance.fr/determinants-de-sante/vaccination/donnees. Accessed 14/03/2020).

In France, in 2017, NTHi represented 75% of invasive Hi isolates while Hif and Hib represented 10 and 7% respectively. However, 50% of the Hib cases were among children < 5 years old and represented 13% of Hi cases among children < 5 years [4]. The proportion of Hib cases among children < 5 years prompted enhancing surveillance of Hib cases and conducting a seroprevalence study to measure Hib antibodies in population before and after changing the vaccination schedule. Enhancing surveillance was also warranted as waning of immune response was reported after vaccination schemes in infants < 1 year with conjugate polysaccharide vaccines against *Neisseria meningitidis* serogroup C as protective titres fell to 36% 18 months after vaccination [5]. Similar observation was reported for Hib [6] and for several serotypes of *Streptococcus pneumoniae* [7]*.* For example, after primary vaccination at 2, 3, and 4 months of age, antibody levels that were measured at 8, and 11 months declined for serotype 4 below the seroprotection level of 0.35 μg/ml [7].

## Methods

### Culture conditions and identification of *H. influenzae*

Surveillance of IHiD was previously reported with a case definition that relies on the detection of Hi in sterile sites. Typing of the isolates is performed at the National Reference Centre for meningococci and Haemophilus influenzae (NRCMHi) that has all permissions to access the samples and the data. Full typing of isolates including whole genome sequencing (WGS) was performed as previously described [4]. Multilocus Sequence Typing (MLST) scheme and sequence types (ST) were extracted from WGS [8]. Genomic analysis was performed using the Bacterial Isolate Genome Sequence Database (BIGSdb) platform on PubMLST [9] and GrapeTree was used to visualise the resulting distance matrices [10]. Data are available on https://pubmlst.org/hinfluenzae/ through filtering by country (France) and serotype (b) to allow retrieving of WGS sequences in FASTA formats as well as the ENA accession numbers.

### ELISA-based concentration of anti-polyribosylribitol phosphate (PRP) IgG

The anti PRP IgG were determined using the Kit according to the instruction of the supplier (IBL INTERNATIONAL, Hamburg Germany. Kit *Haemophilus influenzae* b IgG ELISA).

### Sero-prevalence

We performed ELISA on a collection of 232 residual sera from subjects < 5 years of age that were initially received for diagnosis that is part of the routine primary management of suspected invasive bacterial infections.

### Statistical analysis

The individual IgG concentrations were analysed using GraphPad InStat® version 3.06 (GraphPad Software, San Diego, CA, USA). ELISA titres were compared using the Medians values in the different subgroups and the percentages of subjects achieving the threshold of 0.15 μg/mL and 1 μg/mL. Geometric means of IgG concentrations as well as lower and upper 95% confidence intervals were also calculated for cases of vaccine failure. Data were analysed using the Chi-squared test and Mann-Whitney test was used for nonparametric test. A *P* value of < 0.05 was considered to be statistically significant.

## Results

### Evolution of invasive *H. influenzae* cases

**Fig. 1:**
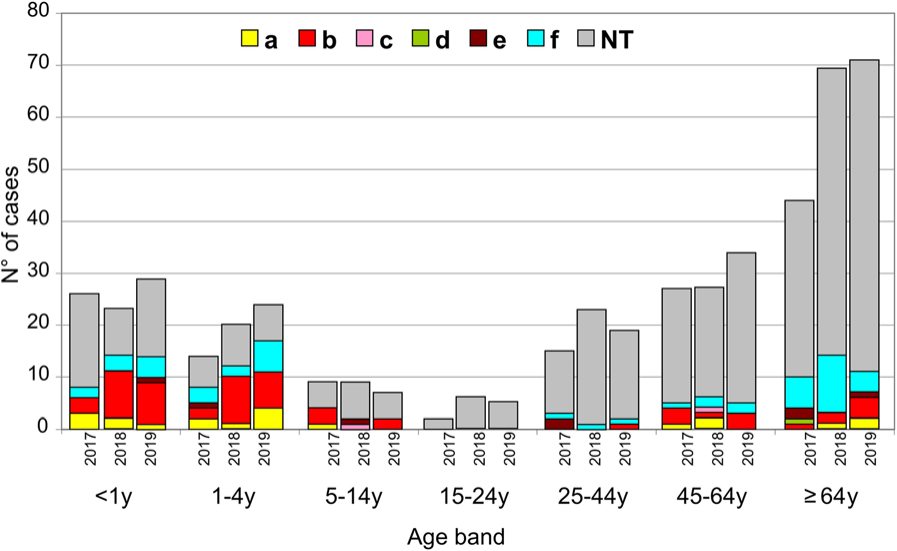
Distribution of Hib invasive cases in France during the period 2017–2019 according to the isolates received at the NRCMHi. Data correspond to number of cases per age and per year

**Fig. 2:**
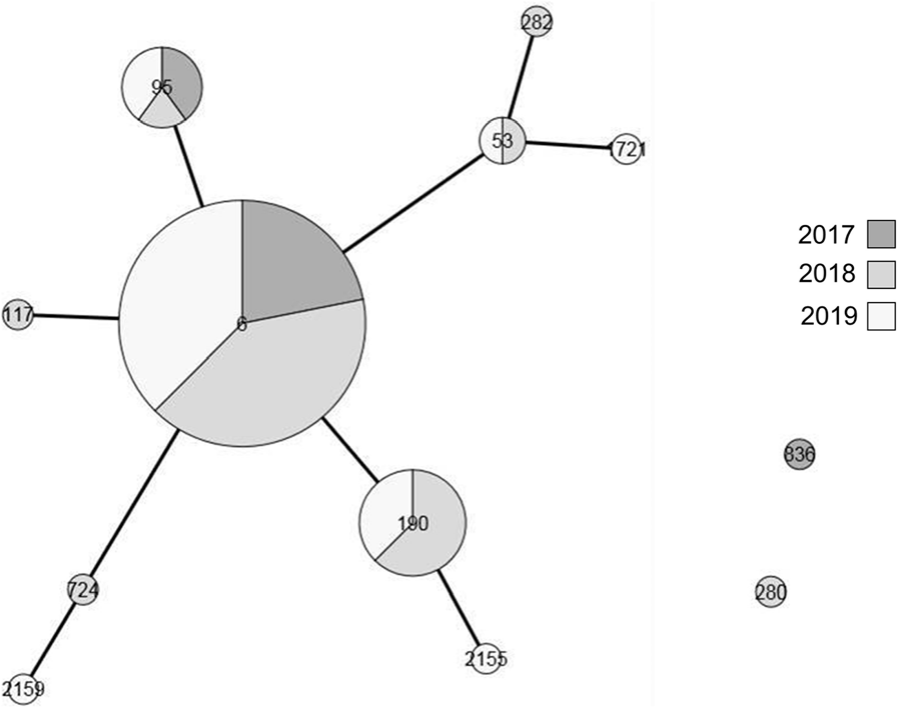
GrapeTree based on the 7 MLST loci from 55 genome of Hib invasive. The nodes were drawn to scale according to the number of isolates and years are indicated by gray colours (indicated by the pie chart) of each node. The ST corresponding to each node was indicated inside the node. The branches between the nodes were drawn to scale only for nodes that differ by no more than two loci

The total number (*n* = 501) of invasive isolates of Hi is depicted in Fig. 1 that showed an increase of the total number between 2017 and 2019. The isolates were of serotype a (*n* = 20; 4%), serotype b (*n* = 56; 11.1%), serotype c (*n* = 2; 0.4%); serotype d (*n* = 1; 0.2%), serotype e (*n* = 8; 1.6%) and serotype f (*n* = 49; 9.7%). The majority of the isolates (*n* = 365; 72.6) were non-typeable. The number of cases due to serotype b isolates increased to reach 25 cases in 2019 with a total number of 56 Hib invasive isolates during this 3-years period. Most of these Hib isolates (*n* = 37; 66.1%) were among children under 5 years of age (median age of 0.8 that ranged between 0.2 years and 4.7 years. This was also observed for Hia invasive isolates with 13 out of 20 isolates (65.0%) among children under 5 years old during the 2017–2019 period. However, Hif and non-typeable invasive isolates were more frequently observed among subjects of 5 years of age and older *n* = 29 out of 49 (59.2%) and *n* = 302 out of 365 (82.7%) respectively. Whole genome sequence (WGS) data were available for 55 of the 56 Hib isolates (98.2%) and showed that most of these isolates belonged to the ST-6 (*n* = 32) and 21 other isolates differed by 1, 2 or three loci from ST-6 (Fig. 2). Indeed, 53 isolates (96% of the sequenced Hib isolates) can then be grouped in one clonal complex (clonal complex ST-6). Only two isolates belonged to two distinct ST (ST-280 and ST-836) that both were detected in adult cases of Hib invasive disease.

### Vaccine failure among Hib cases

The high number (*n* = 37) and proportion (66.1%) of Hib cases among children under 5 years of age during the period 2017–2019 was unexpected. We checked the vaccination status of Hib cases. There were 24 children who were vaccinated completely (*n* = 9) or partially (*n* = 15 with one or two doses) while the remaining 13 did not receive any dose. There was no geographical clustering of vaccine failure cases. The clinical presentations were mainly meningitis (*n* = 16) followed by bacteraemia (*n* = 7) and one case of epiglottitis. All the 24 vaccinated cases were vaccinated according to the new schedule 2 + 1 that was implemented in 2013 in France. They were non premature with no known immunodeficiency. These cases were distributed during this period as 2 cases in 2017 and 11 cases in each 2018 and 2019. We next explored the levels of anti-PRP IgG among the completely vaccinated children and obtained sera at the admission from the 6 corresponding vaccine failures with a complete 2 + 1 scheme.

**Fig. 3:**
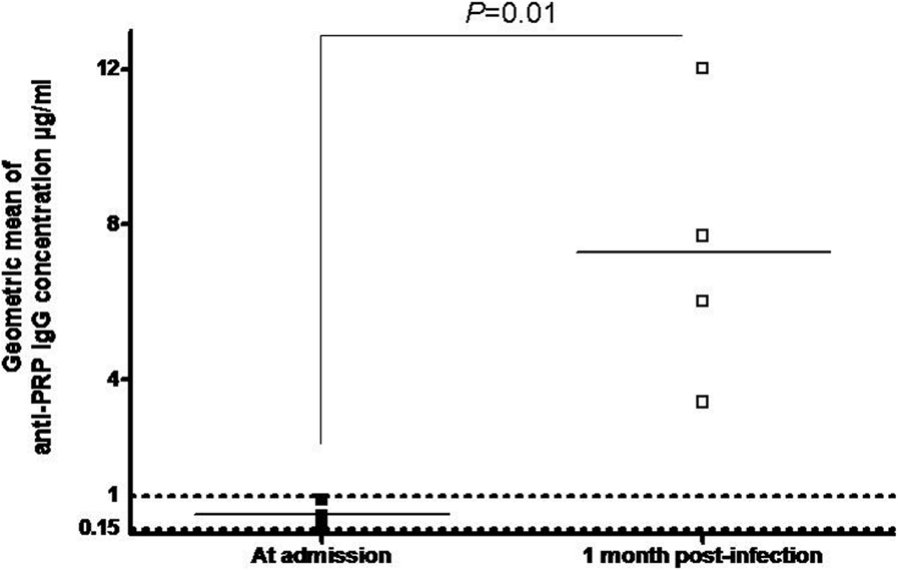
Anti-PRP IgG titres for the 6 cases of vaccine failure among children under the age of 5 years. Titres are given in μg/ml at the admission and 1 month post infection. The data represent each individual tested serum as scattered points around the mean (solid lines). The dotted lines indicate the threshold of 0.15 μg/ml and 1 μg/ml. *P* value was calculated using Mann-Whitney test

The levels of anti-PRP IgG were lower than 1 μg/ml among all the 6 vaccine failure cases (complete 2 + 1 scheme) with median and geometric mean of anti-PRP IgG concentration of 0.43 μg/ml and 0.40 μg/ml (95% CI 0.16–0.98) Respectively (Fig. 3). The median age of these 6 children at the onset of the disease was 2.5 years old and ranged between 1.2 years and 4.1. The median delay between the third dose and the onset of the disease was 1.6 years and ranged between 0.3 and 3.2 years. Sera after 1 month of infection were available from four of these 6 patients with a significantly higher median (6.85 μg/mL) and higher geometric mean of anti-PRP IgG concentration 6.59 μg/ml (95% CI 2.85–15.22; *P* = 0.01), indicated a significant secondary immune response 1 month post infection. All these data suggest that the vaccine failures are most likely due to decline of IgG titres after the third dose at the age of 11 months.

### Sero-prevalence of anti-PRP IgG

**Fig. 4:**
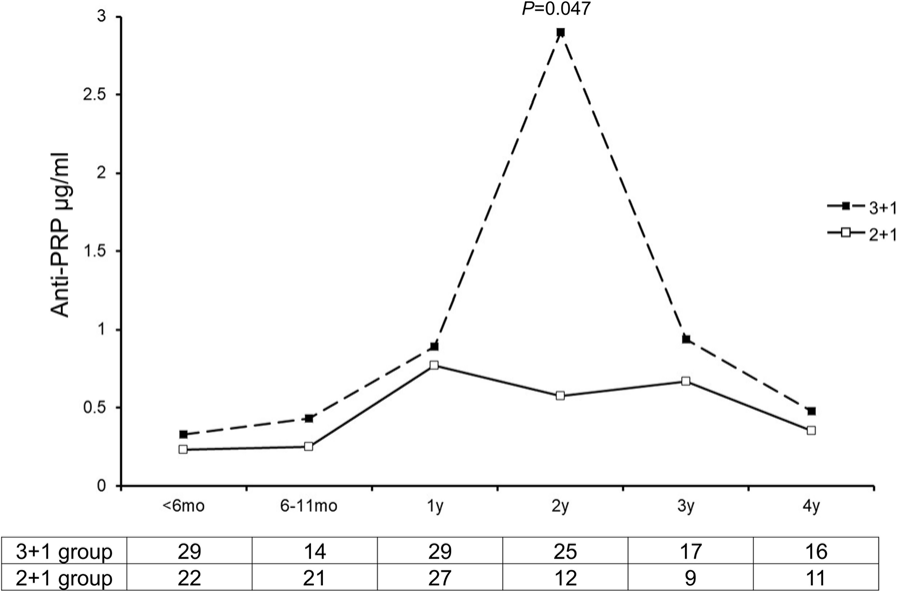
Distribution of values of anti-PRP IgG among children under the age of 5 years. The data are shown as median values per age group and per vaccination schemes (2 + 1 or 3 + 1) as indicated. The number of children in each group is given in the table beneath the Fig. *P* value was calculated using Mann-Whitney test. Only *P* value of < 0.05 is shown for the group of 2 year of age

The observation that all vaccine failures were among children who were immunised according to the 2 + 1 schedule that was introduced since 2013 prompted the exploration of the seroprevalence of anti-PRP IgG among children under the age of 5 years. We used a collection of residual 232 sera that were classified into two groups according to the age: the first group (*n* = 130) was composed of children borne before 2013 and named “3 + 1 group” as this schedule was used before 2013. The second group (*n* = 102) contained the children borne since 2013 and corresponded to the “2 + 1 group”. Each group was further divided into 6 subgroups according to age at the time of sampling: < 6 months, 6–11 months, 1 year, 2 years, 3 years and 4 years of age (Fig. 4).

The median values of anti-PRP IgG were lower in the 2 + 1 group compared to those of the 3 + 1 group in all the 6 subgroups. However, these values did not differ significantly between the corresponding sub-groups except for the two subgroups of children of 2 years of age. The median values of anti-PRP concentrations for children of 2 year of age were 2.9 and 0.58 for the 3 + 1 and 2 + 1 groups respectively (*P* = 0.047) (Fig. 4).

**Fig. 5:**
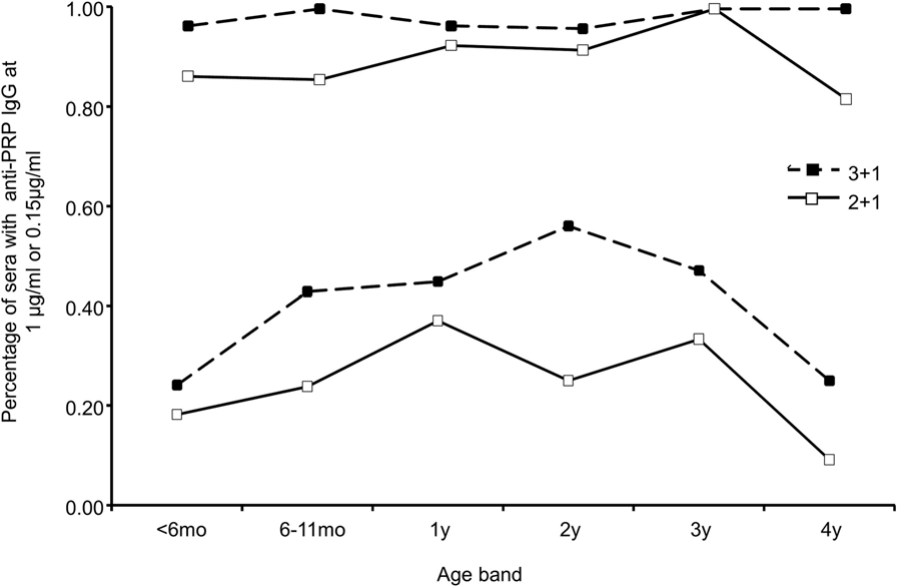
Distribution of percentages of children under the age of 5 years who were vaccinated according to 2 + 1 or 3 + 1 schemes as indicated in the Figure. Sera achieved the threshold of 0.15 μg/ml of anti-PRP IgG are shown by the two curves at the top of the figure. Sera or the threshold of 1 μg/ml are shown in the lower of the figure

The proportion of children who achieved the threshold of 0.15 μg/ml of anti PRP IgG (the putative threshold for short-term protection from invasive Hib disease) were also lower for the 2 + 1 group compared to those of the 3 + 1 group. This was also observed when the threshold was 1 μg/ml of anti-PRP IgG (the putative threshold for long-term protection from invasive Hib disease) and particularly for children of 2 years of age (56% versus 25%) (Fig. 5). These data did not reach significant levels due to small numbers in each subgroup. However, they still suggest that the 3 + 1 group showed higher and more persistent titres of anti-PRP IgG.

## Discussion

Vaccination against Hib allowed the control of Hib infections worldwide [11]. In France, the vaccination in early 1992 resulted in a marked decline of 96% of Hib disease among children aged < 5 years and the incidence declined from 12 to 0.4 /100000 [12]. Hib meningitis became rare among children in France Between 2001 and 2006, with only 36 cases in one series of 2539 cases of bacterial meningitis [13].

However, the recent increase of Hib invasive cases in France observed in this work in spite of high vaccine coverage and the absence of any known risk factor raised several questions. Indeed, all the Hib isolates in children < 5 years of age belonged to the ST-6 or closely linked STs as was already reported at the end of the twentieth century for Hib isolates [8]. This observation suggests that the isolates in our study did not differ from other Hib isolates and particularly in children under the age of 5 years that were all of ST-6 or its derivatives. Our data clearly showed that anti-PRP IgG titres did not peak after a complete 2 + 1 (2, 4 and 11 months) scheme that was launched in 2013 and declined thereafter. At the opposite, the children who were immunised according to the 3 + 1 scheme (2, 3, 4 months and a booster at 16–18 month) showed a peak at the age of 2 years. In parallel, all cases of vaccine failure were observed among children immunised according to the 2 + 1 schemes. High anti-PRP IgG titres are directly associated with protection and lead to a good control of invasive Hib infections as reported in children under 10 years eligible for the 2003 and 2007 campaigns in the United Kingdom [14].

Two thresholds of serum concentration (0.15 and 1.0 μg/ml) of anti-PRP IgG are used to correlate anti-PRP IgG with protection against Hib disease. These thresholds were reported to be correlated with short and long term protections respectively [3]. However, our data from vaccine failure cases argue that the threshold of 0.15 μg/ml is not sufficient for immediate protection. The anti-PRP IgG level of at least 1 μg/ml may be more suitable to predict protection. Our data further showed that the levels of these antibodies after 1 month of infection increased significantly with a geometric mean of concentration of 6.59 μg/ml. This important increase is in favour of a memory-based secondary immune response suggesting that vaccine failures are most likely due to a decline of the immune response in children immunised using the 2 + 1 scheme. The median interval between the completion of the 2 + 1 vaccination and the onset of the disease (1.6 years) is also in favour of “titre decline” hypothesis.

Measuring antibody avidity and the isotype of these antibodies can also add to the understanding of these cases and to refine the hypothesis [3].

## Conclusion

The simplification of anti-Hib vaccination in France in 2013 to a 2 + 1 scheme seems to be associated with lower levels of anti PRP IgG. Hib antibodies also wane rapidly after the 11 months booster and may not be enough to ensure long term protection. Seroprevalence studies as performed in this work are required to inform the decision making in choosing vaccination polices.

## Data Availability

Genomic data are available on https://pubmlst.org/hinfluenzae/. The data that support the findings of this study are available on request from the corresponding author [MKT].

## Abbreviations

IHiD: Invasive *Haemophilus influenzae* disease
Hib: *Haemophilus influenzae* serotype b
MLST: Multilocus sequence typing
PRP: Polyribosylribitol phosphate
WGS: Whole genome sequencing
